# The influence of retardation and dielectric environments on interatomic Coulombic decay

**DOI:** 10.1038/s41467-018-05091-x

**Published:** 2018-07-26

**Authors:** Joshua Leo Hemmerich, Robert Bennett, Stefan Yoshi Buhmann

**Affiliations:** 1 Physikalisches Institut, Albert-Ludwigs-Universität Freiburg, Hermann-Herder-Str. 3, 79104 Freiburg, Germany; 2 Freiburg Institute for Advanced Studies (FRIAS), Albertstr. 19, 79104 Freiburg, Germany

## Abstract

Interatomic Coulombic decay (ICD) is a very efficient process by which high-energy radiation is redistributed between molecular systems, often producing a slow electron, which can be damaging to biological tissue. During ICD, an initially-ionised and highly-excited donor species undergoes a transition where an outer-valence electron moves to a lower-lying vacancy, transmitting a photon with sufficient energy to ionise an acceptor species placed close by. Traditionally the ICD process has been described via ab initio quantum chemistry based on electrostatics in free space, which cannot include the effects of retardation stemming from the finite speed of light, nor the influence of a dispersive, absorbing, discontinuous environment. Here we develop a theoretical description of ICD based on macroscopic quantum electrodynamics in dielectrics, which fully incorporates all these effects, enabling the established power and broad applicability of macroscopic quantum electrodynamics to be unleashed across the fast-developing field of ICD.

## Abstract (web summary)

Interatomic Coulombic Decay is a non-radiative relaxation process between excited systems. Here the authors report a theoretical framework based on macroscopic quantum electrodynamics that shows the role of retardation and an environment in the enhancement or suppression of the ICD rate.

## Introduction

Exactly one century ago, Einstein showed^1^ that the existence of a thermal equilibrium between matter and radiation implies a process by which atoms can indiscriminately release energy—now known as spontaneous emission. Its origin is the coupling of the atom to the quantum electrodynamical vacuum field that permeates all of space, so while being originally thought of as a fundamental atomic property, spontaneous emission can in fact be tuned by placing the emitter in an environment that modifies the vacuum state—between mirrors, for example. This was first pointed out by Purcell^2^, who predicted that the rate of spontaneous decay could be dramatically enhanced by confining an emitter to a small volume. At a similar time, Casimir and Polder^3^ found that the finite speed of light can impact the London-van der Waals force, originally thought of as an instantaneous and fundamental interaction between particles. In both cases, the explanation of a previously known effect in terms of a more fundamental theory, quantum electrodynamics (QED), led to the prediction of new physics, later verified in experiments. This is the blueprint we wish to follow in the present study of interatomic Coulombic decay (ICD)

**Fig. 1:**
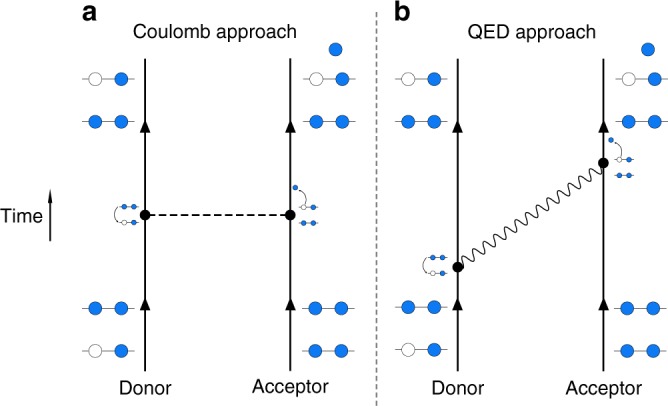
Interatomic Coulombic decay process. Illustration of the interatomic Coulombic decay process in terms of **a** the traditional language of Coulomb interactions and **b** our quantum electrodynamics approach. An ionised, excited atom or molecule (donor) with an inner-valence vacancy (sometimes known as hollow) makes a transition where that vacancy is filled and a photon is emitted. If that photon has an energy higher than the photoionisation threshold of a neighbouring atom or molecule (acceptor), its absorption may be accompanied by an electron being freed into the continuum. The resulting pair of ions then undergoes a ‘Coulomb explosion’, which is one of the experimental signatures of ICD

ICD was first predicted in 1997 by L.S. Cederbaum and coworkers^4^ and experimentally observed shortly afterwards^5–8^. The details of the ICD process are shown in Fig. 1, where in our terminology the interaction-mediating photon is in fact a generalised polariton-like field-matter excitation, containing both radiative (long-range) and non-radiative (short-range) contributions. It is worth noting that the electron left in the continuum is usually of a relatively low energy^9^, as most of the energy the photon transfers to the acceptor is spent freeing the electron. It has been shown that such low-energy electrons can have detrimental effects on biological matter^10^, meaning that ICD is of more than fundamental interest.

Comparison of ICD observations with theory is usually made by elaborate ab initio quantum chemistry approaches^11^—a trend that continues in the recently proposed superexchange^12^, surface-based^13^ and doubly-ionised^14^ ICD variants. These methods are well-suited to dealing with the complex consequences of orbital overlap between donor and acceptor. However, they cannot easily deal with the effects of retardation stemming from the finite speed of light. Furthermore, while a simple dielectric background or extended host molecule can be taken into account with molecular dynamics techniques^15,16^ or via numerical quantum chemistry^17–19^, the full effects of dispersion, absorption, relativistic retardation and sharp boundaries remain computationally beyond reach. This means that such approaches are not able to account for the effects of a complex environment, such as that of a helium nanodroplet^20–23^ or those found in biological settings^10,24,25^.

In the following we present a theoretical description of ICD that systematically includes all of the latter effects, for which we use a framework known as macroscopic QED^26–29^. In this formalism the quantised electromagnetic field in dispersive and absorbing media is described by a dyadic Green’s tensor ${\Bbb G}\left( {{\mathbf{r}},{\mathbf{r}}\prime ,\omega } \right)$ governing propagation of field-matter excitations (medium-assisted photons) of frequency *ω* between a source at position **r**′ and an observation point **r**. Various QED quantities such as spontaneous decay rates and Casimir forces can then be expressed entirely in terms of this Green’s tensor, which is known in closed form for simple geometries^30^ and can be approximated for more complex ones^31^. This allows the computation of such QED quantities in any desired environment, with vacuum emerging as a special case. In this work we use this formalism to show that the range of ICD may be much larger than expected from purely electrostatic considerations, and that an environment such as liquid water can significantly alter the efficiency of ICD. For donor and acceptor situated near a macroscopic body, we predict that the ICD rate can be enhanced or suppressed due to resonant interactions with surface plasmons.

## Results

### Macroscopic QED approach to ICD

Using the formalism of macroscopic QED, we have calculated a general expression for the ICD rate in arbitrary dispersive and absorbing environments, valid at any donor–acceptor distance where orbital overlap can be neglected^32^. We begin with the interaction Hamiltonian of the dipole moments ${\hat{\mathbf d}}_{\mathrm{D}}$of the donor and ${\hat{\mathbf d}}_{\mathrm{A}}$ of the acceptor,

1 $$\hat H_{{\mathrm{int}}} = - {\hat{\mathbf d}}_{\mathrm{D}} \cdot {\hat{\mathbf E}}\left( {{\mathbf{r}}_{\mathrm{D}}} \right) - {\hat{\mathbf d}}_{\mathrm{A}} \cdot {\hat{\mathbf E}}\left( {{\mathbf{r}}_{\mathrm{A}}} \right)$$

where the quantised electric field operator ${\hat{\mathbf E}}{\left( {\mathbf{r}} \right)}$ excites polariton-like field-matter excitations through an appropriate set of bosonic operators. We then evaluate the ICD rate Γ using the interaction Hamiltonian (1) in time-dependent perturbation theory with causal adiabatic coupling. It is important to note that viewing the ICD process as the exchange of virtual photons means that twice as many orders of perturbation theory are needed compared to an instantaneous Coulomb-like picture, as illustrated in Fig. 1.This is because the Coulomb approach considers the donor and acceptor as essentially a single object, which then undergoes ICD as a one-step process (the two interactions in Fig. 1 occur simultaneously). By contrast, the virtual photon approach considers the donor and acceptor completely separately, so that the finite time delay associated with propagation from one to the other is fully taken into account. This doubling of the number of interactions means that in contrast to Coulomb approaches where second order perturbation theory suffices (see, for example ref.^11^), we need to use fourth-order time-dependent perturbation theory. Using the Hamiltonian (1) and expanding the transition matrix element that links our initial and final states in a Dyson series keeping terms up to fourth order in the interaction (see Methods section), we extract the ICD decay rate as;

2 $${\mathrm{\Gamma }} = 2\pi ^2\mathop {\sum}\limits_{{\mathrm{channels}}} \gamma _{\mathrm{D}}\sigma _{\mathrm{A}}(\hbar \omega _{\mathrm{A}}){\mathrm{Tr}}\left[ {{\Bbb G}\left( {{\mathbf{r}}_{\mathrm{A}},{\mathbf{r}}_{\mathrm{D}},\omega _{\mathrm{D}}} \right) \cdot {\Bbb G}^ \ast \left( {{\mathbf{r}}_{\mathrm{D}},{\mathbf{r}}_{\mathrm{A}},\omega _{\mathrm{D}}} \right)} \right],$$

where **r**_A_ and **r**_D_ are, respectively, the positions of the acceptor and donor, *ω*_D_ is the transition frequency of the donor and *ħω*_A_ = *ħω*_D_ − *U*_coul_, where *U*_coul_ is the Coulomb energy of the system. The allowed channels in the above expression are those satisfying *ħω*_D_ ≥ *U*_coul_ + U_ion_, where U_ion_ is the ionisation potential of the acceptor. We make use of the following shorthands; *γ*_D_ is the free-space single-atom decay rate of the donor, given explicitly by

3 $$\gamma _{\mathrm{D}} = \frac{{\omega _{\mathrm{D}}^3\left| {{\mathbf{d}}_{\mathrm{D}}} \right|^2}}{{3\pi \hbar c^3\varepsilon _0}},$$

and *σ*_A_(*E*) is the photoionization cross section of the acceptor

4 $$\sigma _{\mathrm{A}}\left( E \right) = \frac{{\pi E}}{{3\varepsilon _0c\hbar }}\frac{{\mathrm{d}}}{{{\mathrm{d}}E}}\left| {{\mathbf{d}}_{\mathrm{A}}} \right|^2,$$

which is expressed as a function of the energy *E* of the incident photon. We see from equations (3) and (4) that formula (2) contains four dipole moments, each of which corresponds to an interaction vertex in our fourth-order perturbation theory.

Formula (2) can be physically understood as the product of three factors. The first is the free-space decay rate *γ*_D_ of the donor, which is simply a measure of how strongly its coupling to the vacuum causes it to emit, even in the absence of the acceptor or an environment. The second factor is the trace over Green’s tensors, describing the impact of the environment between and around donor and acceptor on the transmission of energy between them. The final factor is the photoionisation cross section *σ*_A_ of the acceptor, evaluated at the photon frequency *ħω*_D_. This factor corresponds to how likely it is that a photon arriving at the acceptor will cause a photoionisation event, freeing an electron from the acceptor. This means the three factors each loosely correspond to a probability of a step in the process (emission, propagation and then absorption), meaning that their product represents an overall rate for ICD.

### Relativistic retardation in ICD

The first consequence of Eq. (2) that we will highlight is the effect of retardation originating from the finite speed of light (note that the related process of two-centre resonant photoionisation has also been shown to be affected by retardation in free space^33^). The consequences of this are expected to be the most dramatic at large donor–acceptor distances—systems with this character have been the focus of recent experimental work^34,35^. Using the vacuum Green’s tensor [see, for example, ref.^29^ or the Methods section] in our formula (2) we find;

5 $${\mathrm{\Gamma }}_{{\mathrm{vac}}} = \frac{1}{4}\mathop {\sum}\limits_{{\mathrm{channels}}} \frac{{\gamma _{\mathrm{D}}\sigma _{\mathrm{A}}\left( {\hbar \omega _{\mathrm{A}}} \right)}}{{\left( {2\pi /\lambda _{\mathrm{D}}} \right)^4\rho ^6}}\left[ {3 + \left( {\frac{{2\pi \rho }}{{\lambda _{\mathrm{D}}}}} \right)^2 + \left( {\frac{{2\pi \rho }}{{\lambda _{\mathrm{D}}}}} \right)^4} \right],$$

where *ρ* = |**r**_D_ − **r**_A_| is the donor–acceptor separation and *λ*_D_ = 2*πc*/*ω*_D_ is the wavelength of the photon emitted from the donor. In the limit of short distances only the first term in the square brackets of (5) remains, this is is the non-retarded vacuum rate ${\mathrm{\Gamma }}_{{\mathrm{vac}}}^{{\mathrm{NR}}}$;

6 $${\mathrm{\Gamma }}_{{\mathrm{vac}}}^{{\mathrm{NR}}} = \frac{3}{4}\mathop {\sum}\limits_{{\mathrm{channels}}} \frac{{\gamma _{\mathrm{D}}\sigma _{\mathrm{A}}\left( {\hbar \omega _{\mathrm{A}}} \right)}}{{\left( {2\pi /\lambda _{\mathrm{D}}} \right)^4\rho ^6}}{\kern 1pt} .$$

**Fig. 2:**
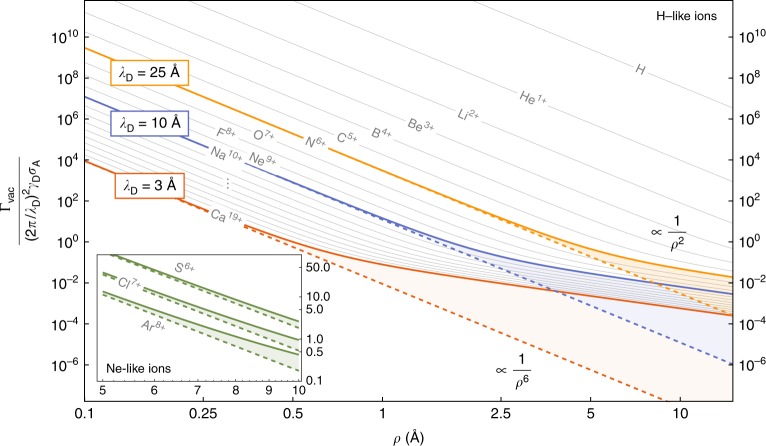
Effect of retardation on interatomic Coulombic decay rate: Main plot: position-dependence of the interatomic Coulombic decay rate for arbitrary acceptor species and a variety of donors, as a function of their mutual separation *ρ*. Shown with thick solid lines are the rates for a selection of donor transition wavelengths *λ*_D_. In the near-field (non-retarded) limit the rate is proportional to *ρ*^−6^ (dashed straight lines), while in the far-field (retarded) limit it becomes proportional to *ρ*^−2^, drastically enhancing the rate relative to that which is found if retardation is ignored. For reference we have shown as thin lines the ICD rates if the donor is taken to be a hydrogen-like ion undergoing a 2P_1/2_ → 2S_1/2_ transition^48^. The rates for the first twenty such ions are shown (labels for Mg^11+^ to K^18+^ are replaced by ‘…’ for formatting reasons) demonstrating that, for example, an ICD process whose donor is a Na^10+^ (*λ*_D_ = 10.05 Å) ion will experience significant retardation at typical ICD distances (3–10 Å). Inset: Same axes as the main plot, instead displaying data relevant to the 1P° → 1S transition in selected neon-like ions

This is the only part of Eq. (5) accessible without using our theory, and as such is reproduced by the more traditional instantaneous Coulomb approach^32,36^, and its inverse sixth power of distance can be understood as coming from the *ρ*^−3^ dependence of the near-field limit of the electric field of a dipole, which is then squared during calculation of dipole–dipole coupling. The second and third terms of Eq. (5) are new and come from the inclusion of the far-field of the dipole which decays as *ρ*^−1^. The new terms are proportional to *ρ*^−4^ and *ρ*^−2^, which obviously means they decay much more slowly at large distances than the *ρ*^−6^ found without including retardation. This can cause dramatic enhancement relative to the rate expected from the non-retarded theory if the condition 2*πρ* > *λ*_D_ is satisfied, as shown in Fig. 2.

Systems undergoing ICD are typically separated by around 3–10 Å, meaning that the condition *ρ* > *λ*_D_/2*π* is comfortably satisfied by, for example, taking Ne^9+^ (nine-fold ionised neon) as a donor species. There the transition energy from the 2P_1/2_ → 2S_1/2_ levels is around 1.02 keV, corresponding to *λ*_D_/2*π* of approximately 1.9 Å.

The importance of retardation is not limited to small, extreme systems such as hydrogen-like ions—even large, multi-electron ions can be near enough to each other to undergo ICD $\left( { \lesssim 10\,{\mathrm{{ \AA}}}} \right)$, while also being far enough for the assumption of negligible orbital overlap to be applicable. The degree of orbital overlap depends strongly on specific choice of donor and acceptor species, so here we refer to the specific example studied in ref.^32^, where it was shown that the effects of orbital overlap become negligible in Ne–Mg ICD if the separation is above ~6–7 Å. In that system the transition wavelength is far too long for retardation to play a role. However, if one replaces the neon donor with a neon-like ion, the transition wavelength will be much shorter, but the degree of orbital overlap should to remain similar, meaning its influence will still become negligible at at about 6–7 Å. We have included in the inset of Fig. 2 the ICD rate for three selected neon-like donors, with arbitrary acceptor species. While one would have to carefully consider the degree of orbital overlap for a neon-like donor and a specific acceptor species in order to decide in which specific distance range our model applies, the preceding discussion and the inset of Fig. 2 together show that ICD between large, multi-electron systems can in principle also be subject to significant retardation corrections.

### ICD in a host medium

**Fig. 3:**
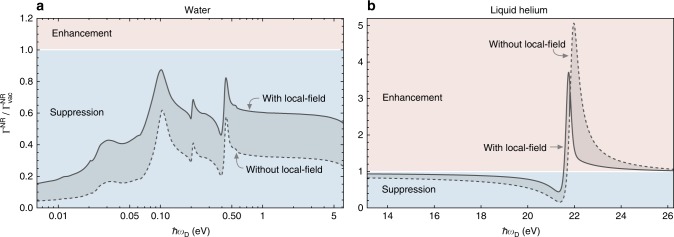
Effect of solvent media on interatomic Coulombic decay rate. Non-retarded interatomic Coulombic decay rate Γ^NR^ in **a** liquid water and **b** liquid helium, using tabulated optical data^40, 41^. The results are normalised to the non-retarded rate $\Gamma _{{\mathrm{vac}}}^{{\mathrm{NR}}}$ encountered in vacuum, given by Eq. (6). As explained in the main text, the bulk medium can enhance or suppress the ICD rate, and the local-field effect can cause an additional suppression or enhancement relative to the results without the local-field effect

The second application of our formula (2) is to ICD processes in a medium. A naive approach to calculating the ICD rate in this situation would be simply changing the optical path length of the photon, but this is not an adequate description of interatomic processes in media, as discussed in ref.^37^. Such an approach misses the fact that an atom embedded in a medium has a small region of empty space around it, meaning the field it experiences is different from that found in the bulk medium—this is known as the local-field effect (LFE). Our approach to ICD can include this by using the appropriate Green’s tensor in Eq. (2), in particular that described in ref.^38^ where the local-field corrected Green’s tensor is calculated for arbitrary media described by a frequency-dependent permittivity *ε*(*ω*). For illustrative purposes we concentrate on the small-distance limit of our formula (2), but we emphasise that our general theory can take into account retardation and medium effects simultaneously. Using the local-field corrected Green’s tensor (see Methods) in the non-retarded limit of our formula (2) we find the rate for a single ICD channel in a bulk medium as;

7 $${\mathrm{\Gamma }}_{{\mathrm{bulk}}}^{{\mathrm{NR}}} = {\mathrm{\Gamma }}_{{\mathrm{vac}}}^{{\mathrm{NR}}} \cdot \eta \cdot \eta _{{\mathrm{LFE}}}\quad \eta _{{\mathrm{LFE}}} = \left| {\frac{{3\varepsilon \left( {\omega _{\mathrm{D}}} \right)}}{{2\varepsilon \left( {\omega _{\mathrm{D}}} \right) + 1}}} \right|^4$$

where *η* = |*ε*(*ω*_D_)|^−2^ is the correction factor that arises in the simple bulk model, *η*_LFE_ is the correction due to the LFE, and we have expressed the rate in terms of ${\mathrm{\Gamma }}_{{\mathrm{vac}}}^{{\mathrm{NR}}}$ as given by Eq. (6). In order to assess the impact of the LFE, we have also shown in Eq. (7) the result that is found if the LFE is ignored (*η*_LFE_ = 1). For quantitative analysis of (7) we have used the measured permittivities for water^40^ and liquid helium^41^, relevant to biological processes and experiments on nanodroplets^22^, respectively. Our results are shown in Fig. 3 where we make a comparison with the rate that would be predicted if LFEs were not taken into account. There we see that inclusion of the LFE can cause enhancement or suppression of the ICD rate, on top of the suppression or enhancement that the simple bulk (that is, without the LFE) causes. The LFE-corrected bulk causes suppression when *η*⋅*η*_LFE_ < 1, which is satisfied when, for example, *ε* < 1 as is the case near the absorption resonance of helium, which is the origin of the enhancement there. For water in the displayed frequency range, the ICD rate is always suppressed by the solvent medium where the neglect of the LFE leads to an overestimation of this suppression. For helium, the LFE significantly narrows the frequency window where the ICD rate is enhanced. The experiment reported in ref.^22^ reveals an ICD signal at 21.6 eV inside a He nanodroplet. Our calculations as shown in Fig. 3 predict an enhancement of the respective ICD rate by a factor 1.7 due to the presence of the bulk nanodroplet medium. To confirm this quantitatively, one has to first use our Eq. (2) in conjunction with the known Green’s tensor of the spherical nanodroplet (see, for example ref.^42^) to calculate the ICD rate for arbitrary donor and acceptor positions with respect to the droplet, a similar study of the effect of a dielectric sphere on the van der Waals interaction between two atoms has been carried out in ref.^43^. Subsequently, the geometric distributions of donors and acceptors, as well as additional decay channels and processes, have to be accounted for to generate a predicted experimental signal. Finally, we note that in this discussion we have concentrated on the effects that the medium has on field propagation between donor and acceptor. In addition, the medium has an impact on the donor and acceptor’s properties such as transition frequencies and dipole moments. This could be studied alternatively using molecular dynamics or macroscopic QED^44^.

### ICD near a dielectric surface

Another demonstration of the power of our general formula (2) is calculation of the effect an inhomogenous environment has on ICD, taking inspiration from the Purcell enhancement of the spontaneous decay rate. Similarly to our presentation of the rates in a bulk medium, we will concentrate on the short-distance limit of our formula (2), but again emphasise that our general theory can simultaneously take into account all the effects discussed so far. Using the dyadic Green’s tensor for a dielectric surface^29,30^ with frequency-dependent permittivity *ε*(*ω*) occupying the region *z* < 0 (see Methods) in our formula (2), we find the following rate in the non-retarded regime;

8 $$	\hskip 60pt{\mathrm{\Gamma }}_{{\mathrm{plate}}}^{{\mathrm{NR}}} = \frac{3}{4}\mathop {\sum}\limits_{{\mathrm{channels}}} \frac{{\gamma _{\mathrm{D}}\sigma _{\mathrm{A}}\left( {\hbar \omega _{\mathrm{A}}} \right)}}{{\omega _{\mathrm{D}}^4/c^4}} \\ 	\times \left\{ {\frac{1}{{\rho ^6}} + 2{\mathrm{Re}}\left[ {R\left( {\omega _{\mathrm{D}}} \right)} \right] \left[ {\frac{1}{{3\rho ^3\bar \rho ^3}} -\frac{{\rho ^2 + \bar \rho ^2}}{{2\rho ^5\bar \rho ^5}}\rho _{||}^2} \right] + \frac{{\left| {R\left( {\omega _{\mathrm{D}}} \right)} \right|^2}}{{\bar \rho ^6}}} \right\},$$

where $\bar \rho = \rho \left( {z_D \to - z_D} \right)$, $\rho _{||}^2 = \left( {x_D - x_A} \right)^2 + \left( {y_D - y_A} \right)^2$ and $R\left( \omega \right) = \left( {\varepsilon \left( \omega \right) - 1} \right)/\left( {\varepsilon \left( \omega \right) + 1} \right)$ is the Fresnel reflection coefficient of the surface. In order to make quantitative predictions we assume a permittivity of the Drude–Lorentz form,

9 $$\varepsilon \left( \omega \right) = 1 - \frac{{\omega _p^2}}{{\omega ^2 - \omega _{\mathrm{T}}^2 + i\gamma \omega }},$$

where *ω*_*p*_ is the plasma frequency, *ω*_T_ is a transition frequency and *γ* is a damping constant. This choice of dielectric function causes the reflection coefficient *R*(*ω*) defined above to exhibit a resonance at a frequency $\omega _{\mathrm{S}} = \sqrt {\omega _{\mathrm{T}}^2 + \omega _p^2/2}$, commonly known as the surface plasmon frequency. In Fig. 4 we display the plate-dependent ICD rate (normalised to that for free space) for a donor at a fixed position near the interface while allowing the position of the acceptor to vary. We choose a range of transition frequencies for the donor, both above and below the resonance frequency of the material. It is seen that the medium-dependent ICD rate has an intricate dependence on both the relative positions of the donor and acceptor, and the relationship between the ICD photon frequency and that of the material resonance. For example, at frequencies below the material resonance and with **r**_A_ − **r**_D_ aligned parallel to the surface, the ICD rate is suppressed by a factor of up to 2. This placement of donor and acceptor could be envisaged in a biological context as two molecules sitting on a cell membrane, our theory predicts that the ICD rate between the two will be slower due to the presence of the membrane. Similarly, if the frequency remains below resonance but now **r**_A_ − **r**_D_ is aligned perpendicular to the surface, the ICD rate can be enhanced by a similar factor. This could arise in a biological setting as an aid to efficient energy transport; our theory predicts that the presence of an interface at the end of a linear arrangement of emitters and absorbers would cause a noticeable enhancement of energy transfer between neighbours.

**Fig. 4:**
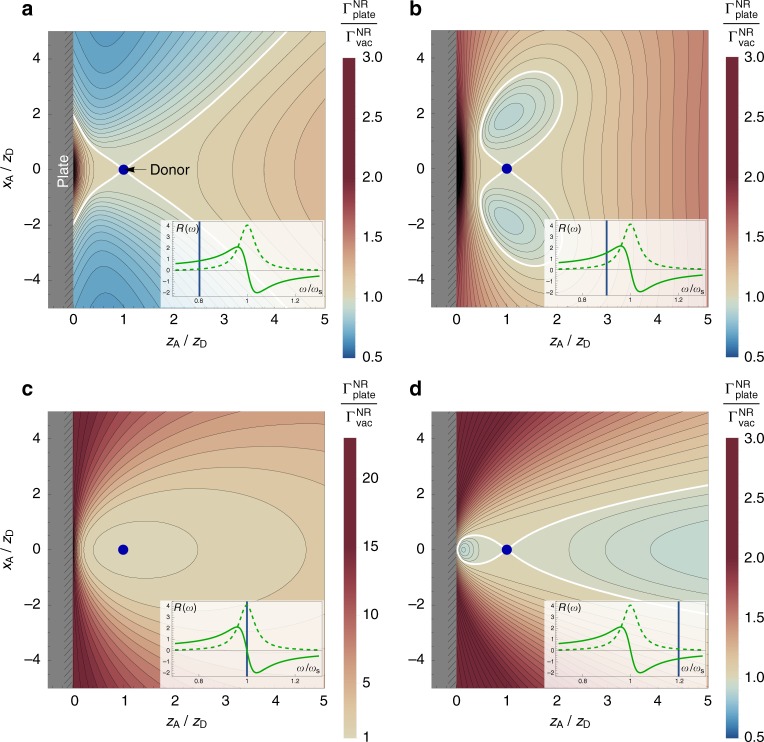
Interatomic Coulombic decay rate near a dielectric surface. Non-retarded interatomic Coulombic decay rate $\Gamma _{{\mathrm{plate}}}^{{\mathrm{NR}}}$ in vacuum near a dielectric surface with Drude–Lorentz permittivity as given by Eq. (9), normalised to the free-space non-retarded rate $\Gamma _{{\mathrm{vac}}}^{{\mathrm{NR}}}$. The donor is at a fixed position on the *z*-axis a distance *z*_D_ from the dielectric, which occupies the region *z* < 0. The acceptor is at position (*x*_A_,0,*z*_A_), which is varied and the rate shown at each position in the *x* − *z* plane. All distances are normalised to *z*_D_, and the Drude–Lorentz parameters are *ω*_*p*_ = *ω*_S_ and *γ* = *ω*_S_/10. The white contours represent the position at which the free-space and surface-modified rates are equal. In the insets we plot the behaviour of the reflection coefficient *R* as a function of frequency near its resonance *ω*_S_, and show with a vertical line the ICD frequency *ω*_D_ chosen in the corresponding plot. The off-resonance plots (**a**, **b** and **d**) share the same scale, while the on-resonance plot (**c**) has a much larger range due to the strong enhancement of the rate if the ICD and material resonance frequencies are equal

Finally, we emphasise that although we have separately presented three ICD-modifying effects (retardation, immersion in a bulk medium and placement near a surface) in order to maintain a clear conceptual divide between each, all of these and more can be taken into account simultaneously in our framework simply by using the appropriate well-known Green’s tensors (c.f. ref.^29^). As an example, we report here the ICD rate found from Eq. (2) for donor and acceptor embedded in an absorbing medium of complex refractive index $\bar n\left( \omega \right) = \sqrt {\varepsilon \left( \omega \right)} = n\left( \omega \right) + i\kappa \left( \omega \right)$, simultaneously including local-field corrections and relativistic retardation. To do this we use the retarded bulk Green’s tensor and the local-field prescription (both of which are found in the Methods section) in Eq. (2), finding;

10 $$	\hskip 50pt{\mathrm{\Gamma }}_{{\mathrm{bulk}}} = \mathop {\sum}\limits_{{\mathrm{channels}}} {\mathrm{\Gamma }}_{{\mathrm{bulk}}}^{{\mathrm{NR}}}e^{ - 2\kappa \rho \omega _{\mathrm{D}}/c}\\ 	\times \left[ {1 + 2\kappa \zeta + \frac{{\zeta ^2}}{3}\left( {4\kappa ^2 + \left| {\bar n} \right|^2} \right) + \frac{{2\kappa \zeta ^3}}{3}\left| {\bar n} \right|^2 + \frac{{\zeta ^4}}{3}\left| {\bar n} \right|^4} \right],$$

where *ζ* = *ρω*_D_/*c*. The material parameters *n*(*ω*) and *κ*(*ω*) are both evaluated at the donor frequency *ω*_D_, and we have used the rate defined by (7) as a shorthand. From Eq. (10) it is evident that there is a complex interplay between the various factors discussed so far, including an exponential screening factor that depends on the extinction coefficient *κ*, as well as polynomial dependence on various combinations of distance and material parameters. In Fig. 5 we plot the separation-dependence of the rate (10) in vacuum and in helium, both with and without local-field corrections. The rates (5) and (7) emerge from Eq. (10) in its vacuum (*n* → 1, *κ* → 0), and non-retarded $\left( {\rho \omega _{\mathrm{D}}/c \ll 1} \right)$ limits, respectively.

**Fig. 5:**
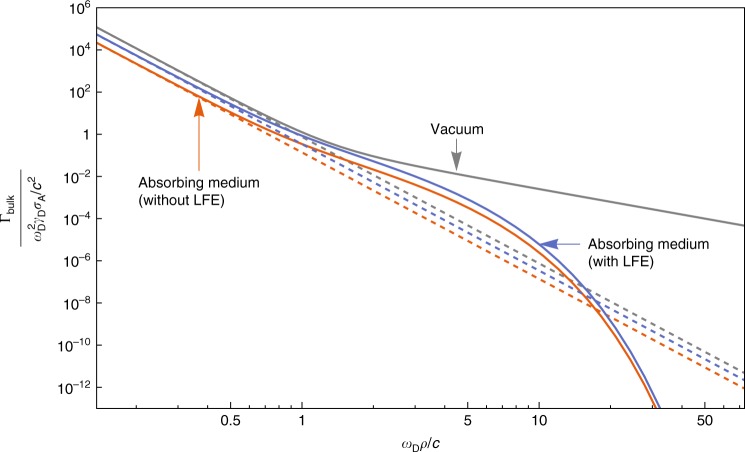
Interplay of medium and retardation effects on interatomic Coulombic decay rate. Shown here is the interatomic Coulombic decay rate for arbitrary donor and acceptor, including medium and retardation effects as given by Eq. (10), as a function of the dimensionless parameter *ω*_D_*ρ*/*c*. The vertical axis is normalised in the same way as that of Fig. 2, and the dashed lines correspond to the non-retarded limits of the three rates shown. The material parameters chosen for the absorbing medium are *n*(*ω*_D_) = 1.49 and *κ*(*ω*_D_) = 0.35, corresponding to the first absorption line of helium^41^

In conclusion, we have presented a description of the ICD rate in arbitrary environments that are described via the electromagnetic Green’s tensor. We have demonstrated some of the explicit consequences of our main result (2), the first being the fundamental importance of retardation which can cause ICD rates to be orders of magnitude higher than expected from any previous theory. We have also studied local-field corrections in a bulk medium, as well as the intricate dependence of the ICD rate upon a nearby macroscopic body. All of these effects should be taken into account when calculating the impact of ICD in the non-idealised, messy situations found in the life sciences, as well as to more fundamental research into medium-dependent ICD. We emphasise that every calculation here proceeded analytically from the same very general formula (2), which was derived using the established power and broad applicability of macroscopic QED, which can now be unleashed across the fast-developing field of ICD.

## Methods

### Derivation of ICD rate

**Fig. 6:**
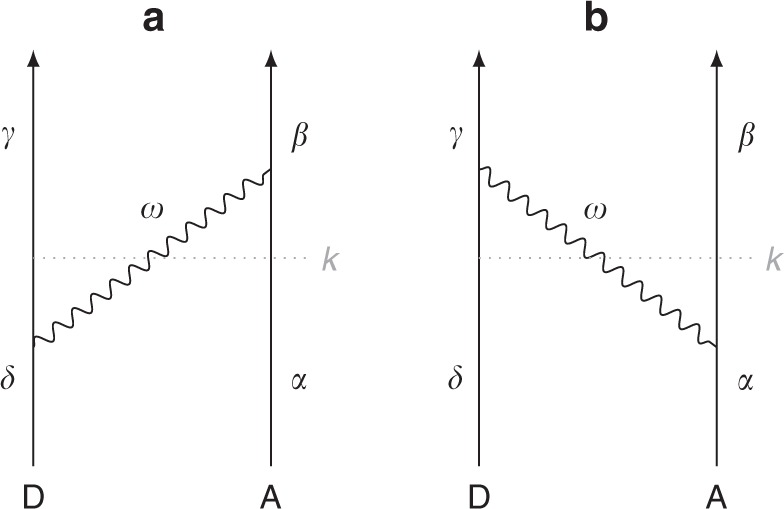
Feynman diagrams for the ICD process. Diagrammatic illustration of the two processes (**a**) and (**b**) supported by our interaction Hamiltonian, for donor D and acceptor A

In this section we derive our central result (2), which is the ICD rate Γ in arbitrary environments. We will calculate Γ from Fermi’s golden rule;

11 $${\mathrm{\Gamma }} = \mathop {\sum}\limits_f \frac{{2\pi }}{\hbar }\left| {M_{fi}} \right|^2\delta \left( {E_i - E_f} \right).$$

where *M*_*fi*_ is a transition matrix element and *E*_*i*_,*E*_*f*_ are the energy eigenvalues of the unperturbed Hamiltonian for the initial and final states |*i*〉 and |*f*〉, respectively. The transition matrix element arising from a time-dependent perturbation $\hat V$ can be calculated using time-dependent perturbation theory. In the causal adiabatic coupling approach (see, for example ref.^45^) one has;

12 $$M_{fi} = \mathop {{\lim}}\limits_{\varepsilon \to 0^ + }\mathop {\sum}\limits_k \frac{{\langle f|\hat V|k\rangle \langle k|\hat V|i\rangle}}{{E_i - E_k + {\mathrm{i}}\hbar \varepsilon }}$$

where *E*_*k*_ is the energy eigenvalue of the intermediate state *k*. In writing Eq. (12) we have assumed that all diagonal elements of $\hat V$ vanish, which is true for the perturbation we shall consider here. The leading-order processes supported by our interaction Hamiltonian (1) are shown in Fig. 6.

We will use *δ*, *γ* and *α*, *β* to represent the states of the donor D and acceptor A, respectively. The donor and acceptor may each be atoms, ions or molecules, though for brevity we describe their states simply as atomic in the rest of this section. In general the states of the acceptor can either be bound or belong to the continuum. During the ICD process the donor decays (*δ* → *γ*), while the acceptor becomes excited (*α* → *β*). In terms of product states |a⟩⊗|b⟩ ≡ |a, b⟩ we can therefore write the ICD process as |*δ*, α⟩ → |*γ*, β⟩. Then the contributions to the sum over intermediate states *k* in Eq. (12) are simply the two possible time-orderings of a process where a single polariton-like field-matter excitation (coinciding with the usual notion of a photon if the process takes place in vacuum) |**1**(**r**, *ω*)⟩ at position **r** with frequency *ω* is exchanged, as shown in Fig. 6. We can hence write the following initial, intermediate and final product states for the composite system of the donor and acceptor coupled to the medium-assisted field whose ground state is |{0}⟩

13 $$\begin{array}{*{20}{l}} {{\mathrm{Initial:}}} \hfill & {|i\rangle = |\delta ,\alpha\rangle| \left\{ 0 \right\}\rangle ,} \hfill & {} \hfill \\ {{\mathrm{Intermediate:}}} \hfill & {|k^{\left( 1 \right)}\rangle = | \gamma ,\alpha\rangle | {\mathbf{1}}\left( {{\mathbf{r}},\omega } \right)\rangle \quad {\mathrm{or}}} \hfill & {k^{(2)} =| \delta ,\beta \rangle|{\mathbf{1}}\left( {{\mathbf{r}},\omega }\rangle \right){\kern 1pt} ,} \hfill \\ {{\mathrm{Final:}}} \hfill & {|f\rangle = |\gamma ,\beta \rangle |\left\{ 0 \right\}\rangle .} \hfill & {} \hfill \end{array}$$

Combining the contributions from both diagrams yields the following expression for the coupling matrix element

14 $$M_{fi} =	 -\mathop {{\lim}}\limits_{\varepsilon \to 0^ + }\mathop {\sum}\limits_{\gamma ,\alpha } {\int} d^3{\mathbf{r}}{\int}_0^\infty d\omega \left[ {\frac{{\langle f |{\hat{\mathbf d}}_{\mathrm{A}} \cdot {\hat{\mathbf E}}\left( {{\mathbf{r}}_{\mathrm{A}}} \right)|k^{\left( 1 \right)}\rangle \langle k^{\left( 1 \right)}|{\hat{\mathbf d}}_{\mathrm{D}} \cdot {\hat{\mathbf E}}\left( {{\mathbf{r}}_{\mathrm{D}}} \right)| i\rangle }}{{\hbar \left( {\omega - \omega _{\delta \gamma } - {\mathrm{i}}\varepsilon } \right)}}}\right. \\ 	+ \left. {\frac{{\langle f|{\hat{\mathbf d}}_{\mathrm{D}} \cdot {\hat{\mathbf E}}\left( {{\mathbf{r}}_{\mathrm{D}}} \right)|k^{\left( 2 \right)}\rangle \langle k^{\left( 2 \right)}|{\hat{\mathbf d}}_{\mathrm{A}} \cdot {\hat{\mathbf E}}\left( {{\mathbf{r}}_{\mathrm{A}}} \right)|i\rangle}}{{\hbar \left( {\omega + \omega _{\beta \alpha } - {\mathrm{i}}\varepsilon } \right)}}} \right],$$

where *ω*_*ab*_ ≡ (*E*_*a*_ − *E*_*b*_)/*ħ* are all positive. We can use the dipole operators ${\hat{\mathbf d}}$ to directly evaluate the atomic part of each term, for example the numerator of the first term becomes;

15 $$	\langle f |{\hat{\mathbf d}}_{\mathrm{A}} \cdot {\hat{\mathbf E}}\left( {{\mathbf{r}}_{\mathrm{A}}} \right)|k^{\left( 1 \right)}\rangle \langle k^{\left( 1 \right)}|{\hat{\mathbf d}}_{\mathrm{D}} \cdot {\hat{\mathbf E}}\left( {{\mathbf{r}}_{\mathrm{D}}} \right)|i\rangle \\ 	= \left( {{\mathbf{d}}_{\alpha \beta } \cdot \langle \left\{ 0 \right\}|{\hat{\mathbf E}}\left( {{\mathbf{r}}_{\mathrm{A}}} \right)|{\mathbf{1}}\left( {{\mathbf{r}},\omega } \right)\rangle } \right)\left( {{\mathbf{d}}_{\delta \gamma } \cdot \langle {\mathbf{1}}\left( {{\mathbf{r}},\omega } \right)|{\hat{\mathbf E}}\left( {{\mathbf{r}}_{\mathrm{D}}} \right)|\left\{ 0 \right\}}\rangle \right)$$

with ${\mathbf{d}}_{\delta \gamma } \equiv \langle \gamma| {\hat{\mathbf d}}_{\mathrm{D}}|\delta \rangle$ and ${\mathbf{d}}_{\alpha \beta } \equiv \langle \beta | {\hat{\mathbf d}}_{\mathrm{A}}|\alpha \rangle$. To evaluate the field-dependent part of each term, we use the definition of the macroscopic QED electric field^26,29^

16 $${\hat{\mathbf E}}\left( {\mathbf{r}} \right) = {\mathrm{i}}{\int}_0^\infty d\omega {\int} d^3{\mathbf{r}}\prime \frac{{\omega ^2}}{{c^2}}\sqrt {\frac{\hbar }{{\pi \varepsilon _0}}{\mathrm{Im}}\varepsilon \left( {{\mathbf{r}}\prime ,\omega } \right)} {\Bbb G}\left( {{\mathbf{r}},{\mathbf{r}}\prime ,\omega } \right) \cdot {\hat{\mathbf f}}\left( {{\mathbf{r}}\prime ,\omega } \right) + {\mathrm{H}}{\mathrm{.c}}{\mathrm{.}}$$

where ${\hat{\mathbf f}}$ and ${\hat{\mathbf f}}^\dagger$ are a set of bosonic operators that create and annihilate combined matter-field excitations^26,29^. For the field evaluated at a general position ***r***_*a*_ we have;

17 $$\begin{array}{*{20}{l}} \langle{\left\{ 0 \right\}|{\hat{\mathbf E}}\left( {{\mathbf{r}}_a} \right)|{\mathbf{1}}\left( {{\mathbf{r}},\omega } \right)}\rangle \hfill & = \hfill & {{\int} d{\kern 1pt} ^3{\mathbf{r}}\prime {\int}_0^\infty d{\kern 1pt} \omega {\mathrm{i}}\frac{{\omega ^2}}{{c^2}}\sqrt {\frac{\hbar }{{\pi \varepsilon _0}}{\mathrm{Im}}\varepsilon \left( {{\mathbf{r}}\prime ,\omega } \right)} {\Bbb G}\left( {{\mathbf{r}}_a,{\mathbf{r}}\prime ,\omega } \right)\delta \left( {{\mathbf{r}} - {\mathbf{r}}\prime } \right)\delta \left( {\omega - \omega^ {\prime}} \right)} \hfill \\ {} \hfill & = \hfill & {{\mathrm{i}}\frac{{\omega ^2}}{{c^2}}\sqrt {\frac{\hbar }{{\pi \varepsilon _0}}{\mathrm{Im}}\varepsilon \left( {{\mathbf{r}} ,\omega } \right)} {\Bbb G}\left( {{\mathbf{r}}_a,{\mathbf{r}},\omega } \right).} \hfill \end{array}$$

Using this result in (14) we find;

18 $$\begin{array}{*{20}{l}} {M_{fi}} = { - \mathop {\sum}\limits_{\gamma ,\alpha } \frac{{\mu _0}}{\pi } \mathop {{\lim}}\limits_{\varepsilon \to 0^ + }\left\{{{\int}_0^\infty d\omega \frac{{\omega ^2}}{{\omega - \omega _{\delta \gamma } - {\mathrm{i}}\varepsilon }}{\int} d^3{\mathbf{r}}\frac{{\omega ^2}}{{c^2}}\mathrm{Im}\varepsilon \left( {{\mathbf{r}},\omega } \right) } \right.} \hfill \\ \times \left[ {{\mathbf{d}}_{\alpha \beta } \cdot {\Bbb G}\left( {{\mathbf{r}}_{\mathrm{A}},{\mathbf{r}},\omega } \right)} \right] \left[ {{\mathbf{d}}_{\delta \gamma } \cdot {\Bbb G}^ \ast \left( {{\mathbf{r}}_{\mathrm{D}},{\mathbf{r}},\omega } \right)} \right] \hfill\\ {} {\left. { + {\int}_0^\infty d{\kern 1pt} \omega \frac{{\omega ^2}}{{\omega + \omega _{\beta \alpha } - {\mathrm{i}}\varepsilon }}{\int} d^3{\mathbf{r}}\frac{{\omega ^2}}{{c^2}}\mathrm{Im}\varepsilon \left( {{\mathbf{r}},\omega } \right)\left[ {{\mathbf{d}}_{\gamma \delta } \cdot {\Bbb G}\left( {{\mathbf{r}}_{\mathrm{D}},{\mathbf{r}},\omega } \right)} \right]\left[ {{\mathbf{d}}_{\alpha \beta } \cdot {\Bbb G}^ \ast \left( {{\mathbf{r}}_{\mathrm{A}},{\mathbf{r}},\omega } \right)} \right]} \right\}.} \hfill \end{array}$$

We can now use the following integral relation for the Green’s tensor^26,29^

19 $$\frac{{\omega ^2}}{{c^2}}{\int} d{\kern 1pt} ^3{\mathbf{s}}\, {\mathrm{Im}}\left[ {\varepsilon \left( {{\mathbf{s}},\omega } \right)} \right]{\Bbb G}\left( {{\mathbf{r}},{\mathbf{s}},\omega } \right) \cdot {\Bbb G}^ \ast \left( {{\mathbf{s}},{\mathbf{r}}\prime ,\omega } \right) = {\mathrm{Im}}\left[ {{\Bbb G}\left( {{\mathbf{r}},{\mathbf{r}}\prime ,\omega } \right)} \right],$$

to carry out the position integral in (18), obtaining

20 $$\begin{array}{*{20}{l}} {M_{fi}} \hfill & = \hfill & { - \frac{{\mu _0}}{\pi }\mathop {{\lim}}\limits_{\varepsilon \to 0^ + } \mathop {\sum}\limits_{\gamma ,\alpha } {\int}_0^\infty {\kern 1pt} d\omega \,\left[ {\frac{{\omega ^2}}{{\omega - \omega _{\delta \gamma } - {\mathrm{i}}\varepsilon }}{\mathbf{d}}_{\delta \gamma } \cdot {\mathrm{Im}}{\Bbb G}\left( {{\mathbf{r}}_{\mathrm{D}},{\mathbf{r}}_{\mathrm{A}},\omega _{\delta \gamma }} \right) \cdot {\mathbf{d}}_{\alpha \beta }} \right.} \hfill \\ {} \hfill & {} \hfill & {\left. { + \frac{{\omega ^2}}{{\omega + \omega _{\alpha \beta } - {\mathrm{i}}\varepsilon }}{\mathbf{d}}_{\delta \gamma } \cdot {\mathrm{Im}}{\Bbb G}\left( {{\mathbf{r}}_{\mathrm{D}},{\mathbf{r}}_{\mathrm{A}},\omega _{\beta \alpha }} \right) \cdot {\mathbf{d}}_{\alpha \beta }} \right]} \hfill \end{array}$$

where we have assumed that the dipole moments are real. Deforming the integration contour into the upper half of the complex frequency plane we pick up a resonant term from a pole at *ω*_*δγ*_ and an off-resonant contribution along the positive imaginary axis. Taking the limit *ε*→0^+^ we find;

21 $$M_{fi} = - \mu _0\mathop {\sum}\limits_{\gamma ,\alpha } {\mathbf{d}}_{\delta \gamma } \cdot \left\{ {\omega _{\delta \gamma }^2\left[ {{\Bbb G}\left( {{\mathbf{r}}_{\mathrm{D}},{\mathbf{r}}_{\mathrm{A}},\omega _{\delta \gamma }} \right) + {\Bbb F}\left( {{\mathbf{r}}_{\mathrm{D}},{\mathbf{r}}_{\mathrm{A}},\omega _{\delta \gamma }} \right)} \right]}\right. \\ -\left.{ \omega _{\beta \alpha }^2{\Bbb F}\left( {{\mathbf{r}}_{\mathrm{D}},{\mathbf{r}}_{\mathrm{A}},\omega _{\beta \alpha }} \right)} \right\} \cdot {\mathbf{d}}_{\alpha \beta }$$

where

22 $${\Bbb F}\left( {{\mathbf{r}},{\mathbf{r}}\prime ,\omega } \right) \equiv {\int}_{\!\ 0}^\infty d\xi \frac{{\omega \xi ^2{\Bbb G}\left( {{\mathbf{r}},{\mathbf{r}}\prime ,{\mathrm{i}}\xi } \right)}}{{\omega ^2 + \xi ^2}}$$

is the off-resonant contribution. This expression is now suitable for substitution into Fermi’s golden rule (11), giving the rate Γ_*δα*_ for one particular choice of initial state |*δ*,α⟩;

23 $${\mathrm{\Gamma }}_{\delta \alpha } =	 \mathop {\sum}\limits_{\beta ,\gamma } \frac{{2\pi }}{{9\hbar }}\mu _0^2\left| {{\mathbf{d}}_{\alpha \beta }} \right|^2\left| {{\mathbf{d}}_{\delta \gamma }} \right|^2\mathrm{Tr}\left[ {{\Bbb G}\left( {{\mathbf{r}}_{\mathrm{A}},{\mathbf{r}}_{\mathrm{D}},\omega _{\delta \gamma }} \right){\Bbb G}^ \ast \left( {{\mathbf{r}}_{\mathrm{D}},{\mathbf{r}}_{\mathrm{A}},\omega _{\beta \alpha }} \right)} \right] \\ 	\times\delta \left( {\hbar \omega _{\delta \gamma } - \hbar \omega _{\beta \alpha }} \right),$$

where we have taken isotropically averaged dipole moments,

24 $${\mathbf{d}} \otimes {\mathbf{d}} = \frac{1}{3}\left| {\mathbf{d}} \right|^2{\Bbb I}$$

where ${\Bbb I}$ is the identity matrix.

In evaluating the sum over final states in (23), one must be mindful of the fact that the transition |*α*〉 → |*β*〉 is a photoionisation process, meaning that its final state is part of the continuum. This means that the relevant observable is the photoionisation cross section *σ*_*α*_ at a certain incident energy *E*, rather than the dipole moment **d**. The two quantities can however be related;^46,47^

25 $$\mathop {\sum}\limits_{\beta \in {\cal C}} \frac{{\mathrm{d}}}{{{\mathrm{d}}\omega }}\left| {{\mathbf{d}}_{\alpha \beta }\left( \omega \right)} \right|^2 = \frac{{3\varepsilon _0c\hbar }}{{\pi \omega }}\sigma _\alpha \left( E \right).$$

Similarly, the continuum nature of the final states means that the formal sum over *β* is in a fact sum over discrete states $\beta \in {\cal D}$ corresponding to distinct ICD channels, as well as an integral over continuum states $\beta \in {\cal C}$ for each individual channel. We use the following rule for converting a sum over discrete states with energies $\hbar {\omega\prime _i}$ to an integral over a continuous variable *ω*′;

26 $$\mathop {\sum}\limits_{\beta \; \in \;{\cal C}} \left| {{\mathbf{d}}_{\alpha \beta }\left( {\omega\prime } \right)} \right|^2f\left( {\omega\prime } \right)\delta \left( {\hbar \omega - \hbar \omega{\prime}} \right) \to	 {\int}_0^\infty \quad {\mathrm{d}}\omega\prime \frac{{\mathrm{d}}}{{{\mathrm{d}}\omega{\prime}}}\frac{{\left| {{\mathbf{d}}_{\alpha \beta }\left( {\omega\prime } \right)} \right|^2}}{\hbar }f\left( {\omega\prime } \right)\delta \left( {\omega - \omega\prime } \right) \\ 	 = \frac{{3\varepsilon _0c}}{{\pi \omega }}\sigma _\alpha \left( {\hbar \omega } \right)f\,\left( \omega \right)$$

where *f*(*ω*) is an arbitrary smooth function. This finally gives;

27 $${\mathrm{\Gamma }}_{\delta \alpha } = 2\pi ^2\mathop {\sum}\limits_\gamma \gamma _{\delta \gamma }\sigma _\alpha \left( {\hbar \omega _{\delta \gamma }} \right)\mathrm{Tr}\left[ {{\Bbb G}\left( {{\mathbf{r}}_{\mathrm{A}},{\mathbf{r}}_{\mathrm{D}},\omega _{\delta \gamma }} \right) \cdot {\Bbb G}^ \ast \left( {{\mathbf{r}}_{\mathrm{D}},{\mathbf{r}}_{\mathrm{A}},\omega _{\delta \gamma }} \right)} \right],$$

with *γ*_*δγ*_ being the free-space decay rate of the donor;

28 $$\gamma _{\delta \gamma } = \frac{{\omega _{\delta \gamma }^3\left| {{\mathbf{d}}_{\delta \gamma }} \right|^2}}{{3\pi \hbar c^3\varepsilon _0}}.$$

In the main text we make use of the fact that the sum in Eq. (27) represents a sum over allowed ICD channels for a given initial state |*δ*,*α*〉, so that we write $\mathop {\sum}\nolimits_{{\mathrm{channels}}}$ instead of $\mathop {\sum}\nolimits_\gamma$. Upon using the shorthands **d**_D_ ≡ **d**_*δγ*_, *ω*_D_ = *ω*_*δγ*_ and Γ_*δα*_ = Γ, and renaming *σ*_*α*_ = *σ*_A_, the derivation of Eq. (2) in the main text is now complete.

### Green’s tensors

In this section we specify the Green’s tensors ${\Bbb G} \left( {{\mathbf{r}},{\mathbf{r}}\prime ,\omega } \right)$ used to derive the ICD rates (5), (7), (8) and (10) in the main text. Subject to appropriate boundary conditions, these are defined to solve;

29 $$\nabla \times \nabla \times {\Bbb G}\left( {{\mathbf{r}},{\mathbf{r}}\prime ,\omega } \right) - \frac{{\omega ^2}}{{c^2}}\varepsilon \left( {{\mathbf{r}},\omega } \right){\Bbb G}\left( {{\mathbf{r}},{\mathbf{r}}\prime ,\omega } \right) = {\Bbb I}\delta ^{\left( 3 \right)}\left( {{\mathbf{r}} - {\mathbf{r}}\prime } \right),$$

where *ε*(**r**, *ω*) is relative permittivity, which in general depends on both position ***r*** and frequency *ω*. Here we denote the 3 × 3 identity matrix as ${\Bbb I}$.

### Bulk medium

A translationally-invariant medium has *ε*(**r**, *ω*) = *ε*(*ω*), for which the Green’s tensor is (see, for example ref.^29^):

30 $$	\hskip 65pt{\Bbb G}^{\left( 0 \right)}\left( {{\mathbf{r}},{\mathbf{r}}\prime ,\omega } \right) = - \frac{{\Bbb I}}{{3k^2}}\delta ^{\left( 3 \right)}\left( {\mathbf{\rho }} \right) \\ 	- \frac{{e^{{\mathrm{i}}k\rho }}}{{4\pi k^2\rho ^3}}\left\{ {\left[ {1 - {\mathrm{i}}k\rho - \left( {k\rho } \right)^2} \right]{\Bbb I} - \left[ {3 - 3{\mathrm{i}}k\rho - \left( {k\rho } \right)^2} \right]{\mathbf{e}}_\rho \otimes {\mathbf{e}}_\rho } \right\},$$

where $k = \left( {\omega /c} \right)\sqrt {\varepsilon \left( \omega \right)}$, **ρ** = **r** −**r**′, *ρ* = |**ρ**|, and **e**_*ρ*_ = **ρ**/*ρ*. The vacuum Green’s tensor ${\Bbb G}_{{\mathrm{vac}}}^{\left( 0 \right)}\left( {{\mathbf{r}},{\mathbf{r}}\prime ,\omega } \right)$ is found from this by setting *ε*(*ω*) = 1.

### Local-field corrections

Local-field corrections can be introduced on the level of the Green’s tensor. Ultimately, the LFE leads to a correction factor for the bulk Green’s tensor, which amounts to a replacement;

31 $${\Bbb G}^{\left( 0 \right)}\left( {{\mathbf{r}},{\mathbf{r}}\prime ,\omega } \right)\quad \to \quad \left( {\frac{{3\varepsilon \left( \omega \right)}}{{2\varepsilon \left( \omega \right) + 1}}} \right)^2{\Bbb G}^{\left( 0 \right)}\left( {{\mathbf{r}},{\mathbf{r}}\prime ,\omega } \right),$$

as discussed in detail in ref.^39^.

### Dielectric half-space

Here we consider a dielectric medium of permittivity *ε*(*ω*) filling the region *z* < 0, with *z* > 0 being vacuum. We are interested in the non-retarded (near-field) limit defined by $\omega \rho /c \ll 1$, for which the half-space Green’s tensor ${\Bbb G}_{{\mathrm{HS}}}\left( {{\mathbf{r}},{\mathbf{r}}\prime ,\omega } \right)$ in the region *z* > 0 can be obtained by an image construction based on the non-retarded vacuum Green’s tensor above. One finds, in agreement with ref.^48^;

32 $${\Bbb G}_{{\mathrm{HS}}}\left( {{\mathbf{r}},{\mathbf{r}}\prime ,\omega } \right) = {{\Bbb G}_{{\mathrm{vac,NR}}}^{(0)}\left( {{\mathbf{r}},{{\mathbf r}}\prime ,\omega } \right) +\frac{{\varepsilon \left( \omega \right) - 1}}{{\varepsilon \left( \omega \right) + 1}}{\Bbb G}_{{\mathrm{vac,NR}}}^{(0)}\left( {{\mathbf{r}},{\bar{\mathbf r}}\prime ,\omega } \right)\cdot \left( {\begin{array}{*{20}{c}} { - 1} & 0 & 0 \\ 0 & { - 1} & 0 \\ 0 & 0 & 1 \end{array}} \right)} ,$$

where $${\bar{\mathbf r}} = \left( {x,y, - z} \right)$$, and

33 $${\Bbb G}_{{\mathrm{vac,NR}}}^{(0)}\left( {{\mathbf{r}},{\mathbf{r}}\prime ,\omega } \right) = \frac{{{\Bbb I}c^2}}{{3\omega ^2}}{{\delta }}\left( {\mathbf{\rho }} \right) - \frac{{c^2}}{{4\pi \omega ^2\rho ^3}}\left( {{\Bbb I} - 3{\mathbf{e}}_\rho \otimes {\mathbf{e}}_\rho } \right)$$

is the non-retarded limit of the vacuum Green’s tensor. Given relations (30), (31) and (32) it is then a matter of algebra to arrive at the ICD rates (5), (7), (8) and (10).

### Data availability

All data contained within the figures are generated solely from the formulae in the manuscript using the given parameters, with the exception of Fig. 3 where optical data from refs.^40,41^ were used. The transition frequencies of hydrogen- and neon-like ions are publicly available in the NIST Atomic Spectra Database^37^.
